# Editorial: Opioids in the time of the COVID-19 pandemic: from cellular mechanisms to public health policy

**DOI:** 10.3389/fphar.2023.1251958

**Published:** 2023-07-14

**Authors:** Scott John, David R. Walwyn, Kathryn DeFea, Tim G. Hales, Wendy Walwyn

**Affiliations:** ^1^ UCLA Health System, University of California Los Angeles, Los Angeles, CA, United States; ^2^ Department of Engineering and Technology Management, University of Pretoria, Pretoria, South Africa; ^3^ Division of Biomedical Sciences, University of California Riverside, Riverside, CA, United States; ^4^ School of Medicine, University of Dundee, Dundee, United Kingdom

**Keywords:** opioid use disorder, substance use disorder, COVID-19, opioid use disorder treatment, substance use disorder treatment

With hindsight our understanding of the COVID-19 global pandemic caused by the SARS-CoV2 virus, its mutations and related illnesses, has improved (Faust et al., 2021). Sadly, this pandemic coincided with an escalating opioid epidemic that was already reacting to an increased regulation of prescription opioids and turning to a more deadly option, fentanyl and its derivatives. Although difficult to establish causality between these two global “events,” it is clear that there were a greater number of deaths from drug and opioid overdose during the pandemic (Faust et al., 2021; Ghose et al., 2022). This link is further explored by Hutchison et al., who found that opioid-induced poisoning and presentation at emergency care increased in line with each phase of the pandemic. Despite this increase, there was a concomitant decrease in the presentation of Opioid Use Disorder (OUD), possibly a result of treatments and diagnoses not being initiated or continued as the medical teams focused on treating COVID-19 patients. Adding to the problem of the lack of available care, the pandemic posed considerable challenges to harm reduction and substance use treatment. This was highlighted by Radfar et al., who showed from a survey of 77 countries, that the supplies of drugs, buprenorphine and methadone, used to treat Substance Use Disorder (SUD) was impacted in almost half of these countries. Also impacting the SUD patients during the pandemic was a psychological vulnerability, manifest as an increase in negative emotions and poor self-concept, or negative affect (NA), in those over 50, in particular females, with SUD, Wang et al. These authors also showed that, in these patients, the degree of NA was positively correlated with the degree of drug use frequency, craving and also impulsivity. This study outlines the vulnerability of older SUD patients during a pandemic that may be associated with social isolation induced by countries around the world to curb the spread of the virus. Fuchs-Leinter et al. added another dimension to the effect of COVID-19 on mental health and surveyed a clinical sample of patients in Opioid Substitution Therapy (OST) for Post-Traumatic Stress Disorder (PTSD) in Austria. Using a scale specifically adapted to assess PTSD symptoms due to the COVID-19 pandemic, these authors found that 27% of OST patients appeared to be at an elevated risk for PTSD with those at highest risk more likely to show increased craving and also greater depression, anxiety and stress. Putting such mental and life stress factors as risk factors into a measurable scale showed a positive correlation with the risk of fatal or non-fatal overdose (Doggui et al.)adding a possible link between the mental health stressors of the pandemic with increased opioid harms of the time.

SUD/OUD patients were also at higher risk of COVID-19 infection and negative health outcomes due to the impact of the virus and repeated exposure to abused substances, particularly opioids, on respiratory and cardiovascular systems (Wang et al., 2021). This is further explored by the work of Arab et al., who performed a post-mortem study of those suffering from OUD in Scotland and found that the evidence of cardiovascular disease positively correlated with the presence of opioids in the bloodstream. Interestingly there was also a positive correlation between cardiovascular inflammation and opioid presence in blood, possibly from upregulated inflammatory cytokines (Reece, 2012; Lu et al., 2019). This effect could work in tandem with the “inflammatory storm” well known to negatively impact the outcome of COVID-19.

Another effect of COVID-19 was the initial change in the supply, pricing and use of illicit substances (Mutter et al., 2023) and a continuing shift away from the abuse of prescription opioids in the early days of the pandemic (Castilloux et al., 2023). Weng et al., mined data from the National Health Interview Survey and showed that patients in the United States with cardiac conditions reduced their use of prescription opioids to relieve acute pain. This could reflect both the emphasis by medical teams in treating COVID-19 patients coupled with the reluctance of these patients to seek care during this initial stage of the pandemic. The need for opioids to treat pain is highlighted by Palamin et al., who describe a necessity to use these medications in the Brazilian healthcare system that should be implemented with care given the effects of both opioids and COVID-19 on respiratory and cardiac function.

Against this backdrop of not seeking care or limited care being available, has been the drive by healthcare teams to put protocols in place that could be used during such a pandemic. These protocols would maintain/improve access to care while protecting the ‘frontline’ workers addressing the pandemic. Teck et al., presented five case studies of the use of buprenorphine micro-dosing while transferring patients to a long-acting depot buprenorphine that could be used for a broader range of patients when access to healthcare may be limited. More work is needed to examine this approach but the initial case studies show how such a protocol may be used where traditional approaches such as inpatient detoxification are not feasible. Soyka used a literature review to examine this approach further focusing on transferring patients from methadone, a high efficacy opioid receptor agonist, to buprenorphine, a partial opioid receptor agonist while minimizing withdrawal. This study concluded that buprenorphine microdosing during methadone treatment allows a reduction in methadone administration, a novel approach worthy of further study.

Whilst the pandemic was initially associated with multiple areas of misinformation and broad misconception, less stigma was attached to those suffering from COVID-19 as those suffering from OUD (Okobi et al.). This is an interesting dichotomy that may relate to an implicit bias against those with SUD/OUD compared to those with a transmissible disease. This study, in addition to the study by Guo et al., that examined the perceived effect of lockdown in Wuhan in China on viral transmission in other cities, has highlighted areas of public confidence, or lack thereof, and perceptions, that may have abated over the course of the pandemic.

Over time we have learned much about the pandemic, the policies and systems that will be needed for future scenarios, and, as outlined by Radfar et al., the actions of policymakers and healthcare organizations required to generate business continuity plans. These will ideally maintain and strengthen harm reduction approaches and other provisions needed for the safety and support of SUD/OUD patients at all times.
